# Adverse Metabolic Response to Regular Exercise: Is It a Rare or Common Occurrence?

**DOI:** 10.1371/journal.pone.0037887

**Published:** 2012-05-30

**Authors:** Claude Bouchard, Steven N. Blair, Timothy S. Church, Conrad P. Earnest, James M. Hagberg, Keijo Häkkinen, Nathan T. Jenkins, Laura Karavirta, William E. Kraus, Arthur S. Leon, D. C. Rao, Mark A. Sarzynski, James S. Skinner, Cris A. Slentz, Tuomo Rankinen

**Affiliations:** 1 Human Genomics Laboratory, Pennington Biomedical Research Center, Baton Rouge, Louisiana, United States of America; 2 Departments of Exercise Science and Epidemiology/Biostatistics, University of South Carolina, Columbia, South Carolina, United States of America; 3 Preventive Medicine Laboratory, Pennington Biomedical Research Center, Baton Rouge, Louisiana, United States of America; 4 Department of Kinesiology, University of Maryland, College Park, Maryland, United States of America; 5 Department of Biology of Physical Activity, University of Jyväskylä, Jyväskylä, Finland; 6 Department of Medicine, Duke University Medical Center, Durham, North Carolina, United States of America; 7 School of Kinesiology, University of Minnesota, Minneapolis, Minnesota, United States of America; 8 Division of Biostatistics, Washington University School of Medicine, St. Louis, Missouri, United States of America; 9 Professor Emeritus of Kinesiology, Indiana University, Bloomington, Indiana, United States of America; Tulane School of Public Health and Tropical Medicine, United States of America

## Abstract

**Background:**

Individuals differ in the response to regular exercise. Whether there are people who experience adverse changes in cardiovascular and diabetes risk factors has never been addressed.

**Methodology/Principal Findings:**

An adverse response is defined as an exercise-induced change that worsens a risk factor beyond measurement error and expected day-to-day variation. Sixty subjects were measured three times over a period of three weeks, and variation in resting systolic blood pressure (SBP) and in fasting plasma HDL-cholesterol (HDL-C), triglycerides (TG), and insulin (FI) was quantified. The technical error (TE) defined as the within-subject standard deviation derived from these measurements was computed. An adverse response for a given risk factor was defined as a change that was at least two TEs away from no change but in an adverse direction. Thus an adverse response was recorded if an increase reached 10 mm Hg or more for SBP, 0.42 mmol/L or more for TG, or 24 pmol/L or more for FI or if a decrease reached 0.12 mmol/L or more for HDL-C. Completers from six exercise studies were used in the present analysis: Whites (N = 473) and Blacks (N = 250) from the HERITAGE Family Study; Whites and Blacks from DREW (N = 326), from INFLAME (N = 70), and from STRRIDE (N = 303); and Whites from a University of Maryland cohort (N = 160) and from a University of Jyvaskyla study (N = 105), for a total of 1,687 men and women. Using the above definitions, 126 subjects (8.4%) had an adverse change in FI. Numbers of adverse responders reached 12.2% for SBP, 10.4% for TG, and 13.3% for HDL-C. About 7% of participants experienced adverse responses in two or more risk factors.

**Conclusions/Significance:**

Adverse responses to regular exercise in cardiovascular and diabetes risk factors occur. Identifying the predictors of such unwarranted responses and how to prevent them will provide the foundation for personalized exercise prescription.

## Introduction

Physical activity level and cardiorespiratory fitness are strongly and inversely associated with the risk of cardiovascular-, metabolic-, and aging-related morbidities, as well as premature mortality [1]. To alleviate the health burden associated with sedentary behavior and poor fitness, public health recommendations are that adults be physically active at a moderate intensity for 150 minutes per week or at a vigorous intensity for 75 minutes per week [2].

However, there is considerable interindividual variability in the ability to improve one's cardiorespiratory fitness and cardiometabolic and diabetes risk factor profile in response to regular exercise. This clear finding of the HERITAGE Family Study has been replicated [3], [4], [5], [6]. A fundamental question is whether there are individuals who experience one or several adverse responses (ARs) in terms of exercise-induced changes in common risk factors. This issue is addressed herein based on data from six exercise intervention studies, with a focus on exercise-induced changes in resting systolic blood pressure (SBP), fasting insulin (FI), HDL-cholesterol (HDL-C), and triglycerides (TG). The studies used for this purpose are: HERITAGE Family Study (HERITAGE), DREW, INFLAME, STRRIDE, University of Maryland Gene Exercise Research Study (MARYLAND), and University of Jyväskylä study (JYVASKYLA).

## Methods

Data on a maximum of 1687 adults from six studies were available for analysis. These studies will be briefly described, followed by the definition of AR and the statistical procedures employed. More information on each study is available in Information S1.

### HERITAGE (Health, Risk Factors, Exercise Training And Genetics) Family Study

The sample, study design, and exercise training protocol of HERITAGE have been described elsewhere [7]. Briefly, 473 adults from 99 families of Caucasian descent and 250 Blacks from 105 families or sibships completed the 20-week endurance training program. Parents were 65 years of age or less while offspring ranged in age from 17 to 41 years.

### Dose Response to Exercise in Women (DREW) Study

A complete description of the DREW design and methods and details of the study participants have been published [8]. In brief, it was a randomized, dose-response exercise trial with sedentary, high-normal blood pressure, postmenopausal, overweight or obese women (N = 326: 63% White) assigned to either a nonexercise control group or to endurance exercise groups that expended 4, 8, or 12 kcal/kg of body weight per week for a period of 6 months [6].

### Inflammation and Exercise (INFLAME) Study

Sedentary men and women between the ages of 30 and 75 years who had an elevated plasma C-reactive protein (CRP) concentration (≥2.0 mg/L but <10.0 mg/L) were randomized to an endurance exercise or a control group [9]. Completers (70% Whites) exercised a mean of 204 minutes per week.

### Studies of a Targeted Risk Reduction Intervention through Defined Exercise (STRRIDE)

STRRIDE (84% Whites) includes two complementary studies [10], [11]. STRRIDE was composed of 40- to 65-year-old, sedentary, overweight or class 1 obese (BMI 25–35 kg/m^2^), dyslipidemic men and women. They were assigned to one of three aerobic exercise groups and exercised for 6 months. The STRRIDE aerobic training versus resistance training (AT/RT) cohort was very similar to STRRIDE, but only those who were enrolled in endurance exercise programs are included in the present report.

### University of Maryland Gene Exercise Research Study (MARYLAND)

Briefly, 160 men and women (100% Whites) ages 50 to 75 years who were sedentary, nondiabetic, and nonsmoking, with no prior history of cardiovascular disease but with one National Cholesterol Education Program lipid abnormality or blood pressure in the prehypertensive range, exercised three times per week for a period of 6 months [12].

### University of Jyväskylä Study (JYVASKYLA)

Healthy, sedentary 40- to 67-year-old men and women were recruited [13]. A total of 206 subjects were randomized to one of four groups. Here we used the data on 25 men and 26 women of the endurance training group and on 30 men and 24 women (all Whites) of the combined endurance and strength training group who exercised for 21 weeks.

### Definition of adverse responses

For the four traits studied, some subjects experienced changes in an opposite, unfavorable direction compared to the expected beneficial effects. This is analogous to an AR pattern. Defining an AR for any given risk factor is a challenge. A robust definition takes into account the measurement error of the trait, including the variance among laboratories or laboratory technicians, and the normal day-to-day biological variation of the trait. The parameter that captures the totality of these sources of variance in a trait is known as the technical error (TE), defined as the within-subject standard deviation as derived from repeated measures (or assays) over a given period of time, as used in the National Health and Nutrition Examination Survey (NHANES) [14]. An ancillary study designed to quantify TE for several biological traits was undertaken in HERITAGE. Sixty subjects were measured three times (except for FI) over a period of 3 weeks for each trait [15], [16], [17], [18], [19]. TEs and other useful indicators of reproducibility are shown in Table 1. In the case of FI, the assays were performed only twice, and we used other HERITAGE data plus observations from the literature to develop an estimate of TE for FI (Information S1). Here, we have conservatively defined an AR as a response beyond 2×TE in a direction indicating a worsening of the risk factor. For the four traits in the present study, twice the value of TE would mean that ARs would be reached if the exercise training-induced increases are ≥10 mm Hg for SBP, ≥0.42 mmol/L for plasma TG, and ≥24 pmol/L for plasma FI or if there is a decrease of ≤0.12 mmol/L for HDL-C. These AR definitions are used in the remainder of this report.

**Table 1:** Reproducibility of Risk Factors from Measurements Repeated Over 3 Days on 60 Subjects.

Variable	Mean ± SD (at first test)	CV	ICC	TE
Stature, cm	171.7±8.3	0.2	1.00	0.3
Body weight, kg	71.5±12.8	0.9	1.00	0.7
Fasting insulin*, pmol/L	65.8±40.0	19–29	0.78–0.94	13.2–19.8 (12)
HDL-C, mmol/L	1.08±0.25	6.0	0.94	0.06
Triglycerides, mmol/L	1.04±0.47	21.8	0.79	0.21
Systolic BP, mm Hg	118.7±10.3	4.1	0.76	4.9

ICC = intraclass correlation computed from the within-subject variance compared to the overall measurement variance.

TE = technical error defined by the within-subject standard deviation calculated from repeated measurements. It includes a combination of measurement error plus day-to-day variation.

CV = Coefficients of variation is expressed as a percentage and is derived from the technical error and the measurement mean.

* Note on insulin: The values reported here are from the repeated measurements obtained at baseline (N = 779) and after (N = 624) the exercise program in HERITAGE (Information S1). The TE used for this report is shown in parentheses.

To convert pmol/L of insulin to mU/L, divide by 6.945. To convert mmol/L of HDL-C to mg/dL, divide by 0.02586. To convert mmol/L of triglycerides to mg/dl, divide by 0.01129.

### Statistical procedures

Data are expressed as means and standard deviations or standard errors as specified. Intraclass correlations were computed from the within-subject variance relative to the overall measurement variance. The coefficient of variation is expressed as a percentage and is derived from the TE relative to the measurement mean. The significance of the gains in VO_2_max and of the mean changes in the four targeted risk factors within each cohort was assessed with paired t tests. The comparisons of VO_2_max gains between adverse responders and non-adverse responders for each risk factor trait for each study was undertaken as follows: The difference between the changes in VO_2_max with exercise training expressed in ml O_2_ per minute was tested with the general linear model and is reported as least squares (LS) means with age, sex, and baseline VO_2_max as covariates. The gain in VO_2_max % is reported as LS means with age and sex as covariates.

## Results

Subjects in DREW, INFLAME, STRRIDE, MARYLAND, and JYVASKYLA were about 20 years older than HERITAGE Whites and Blacks (Table 2). All cohorts had a mean BMI in the overweight range (i.e., >25.0 but <30.0 kg/m^2^), with the exception of DREW and INFLAME, with mean values of about 31 kg/m^2^. Mean baseline VO_2_max was considerably lower in DREW and INFLAME compared to the other studies. The mean increase in VO_2_max (ml O_2_ per minute) ranged from 108 (DREW) to 395 (HERITAGE Whites). The percent increase of VO_2_max ranged from 8.7% (DREW) to 18.9% (HERITAGE Blacks).

**Table 2:** Descriptive Data, Including Baseline 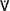
O_2_max and its Response to Training, for the Six Cohorts.

	HERITAGE Whites	HERITAGE Blacks	DREW	INFLAME	STRRIDE	MARYLAND	JYVASKYLA
Maximum number of subjects	473	250	326	70	303	160	105
Age, yrs	35.8 (14.5)	33.6 (11.5)	57.9 (6.5)	51.2 (10)	51.0 (7.7)	58.0 (5.8)	53.5 (7.6)
Baseline BMI, kg/m^2^	25.8 (4.9)	27.8 (5.8)	31.5 (3.9)	31.1 (4.3)	29.9 (2.9)	28.3 (4.6)	25.4 (3.1)
Baseline 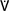 O_2_max, mL/min	2458 (740)	2086 (629)	1312 (240)	1629 (567)	2466 (694)	2060 (536)	2262 (616)
Baseline 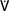 O_2_max, mL/kg/min	33.2 (8.9)	27.3 (7.3)	15.8 (2.5)	19.0 (5.6)	28.2 (6.0)	25.3 (4.6)	29.8 (6.2)
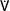 O_2_max response, mL/min	395 (215)	362 (171)	108 (132)	204 (213)	281 (273)	250 (228)	259 (223)
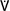 O_2_max response, %	16.9 (9.0)	18.9 (10.3)	8.7 (10.5)	14.1 (13.5)	12.0 (12.0)	12.3 (10.1)	13.0 (11.7)

Values are given as mean (SD). 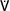
O_2_max response = post-training 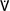
O_2_max minus baseline 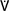
O_2_max (positive value represents improvement in 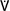
O_2_max).

All gains in VO_2_max are significant at p<0.05.

Baseline values and the mean (±SD) changes of the risk factors in response to exercise programs are shown in Table 3 for each cohort. There was a wide range of baseline values for all risk factors. For instance, mean baseline HDL-C levels ranged from 1.04 mmol/L (HERITAGE Whites) to about 1.50 mmol/L (INFLAME and all DREW exercise groups). The mean changes induced by the exercise programs were generally in the expected direction (i.e., decreases in FI, TG, and SBP and increases in HDL-C). There were, however, some statistically nonsignificant exceptions to these general trends.

**Table 3:** Baseline and training-induced changes in the four risk factors for the five cohorts (mean ± SD).

	HERITAGE		DREW			INFLAME	STRRIDE	Maryland	Jyvaskyla
Variable	Whites (n≤473)	Blacks (n≤250)	4 kcal/kg/wk (n≤143)	8 kcal/kg/wk (n≤89)	12 kcal/kg/wk (n≤94)	(n≤70)	(n≤303)	(n≤160)	(n≤105)
Baseline fasting insulin, pmol/L	65.7±40.0	79.7±63.2	74±41.24	75.85±42.34	70.93±41.08	82.30±40.77	−65.3±41.8	83±31	31.6±16.7
Change in fasting insulin, pmol/L	−5.2±24.9†††	−10.8±44.6†††	−2.02±31.06	−7.98±27.59†	−1.95±29.54	−5.58±31.33	−11.6±29.1†††	−11±21†††	−3.2±14.0
Baseline HDL-C, mmol/L	1.04±0.26	1.09±0.32	1.50±0.38	1.49±0.40	1.50±0.35	1.50±0.39	1.17±0.35	1.24±0.41	1.28±0.40
Change in HDL-C, mmol/L	0.04±0.12†††	0.03±0.13†††	−0.01±0.21	−0.01±0.21	−0.04±0.20	−0.05±0.14††	0.04±0.16	0.08±0.21†††	0.01±0.21
Baseline Tg, mmol/L	1.38±0.78	1.04±0.62	1.45±0.67	1.47±0.68	1.44±0.81	1.28±0.56	1.72±0.89	1.67±1.08	1.19±0.71
Change in Tg, mmol/L	−0.02±0.42	−0.03±0.41	−0.08±0.47	−0.02±0.50	0.03±0.56	0.00±0.46	−0.24±0.64††	−0.21±0.74†††	−0.11±0.54†
Baseline SBP, mm Hg	116.2±10.9	122.8±12.0	138.9±13.4	139.9±13.6	138.5±12.7†	131.3±20.4	N/A	133 + 16	131.7±15.6
Change in SBP, mm Hg	0.2±6.2	−1.2±7.8†	1±12.7	−1.6±15.1	−3.1±11.8	−4.3±13.8†	N/A	1 + 13	−3.7±10.9††

† p≤0.05.

†† p<0.01.

††† p<0.001 indicates significant change score within a group.

To convert pmol/L of insulin to mU/L, divide by 6.945. To convert mmol/L of HDL-C to mg/dL, divide by 0.02586. To convert mmol/L of triglycerides to mg/dl, divide by 0.01129.

**Figure 1:**
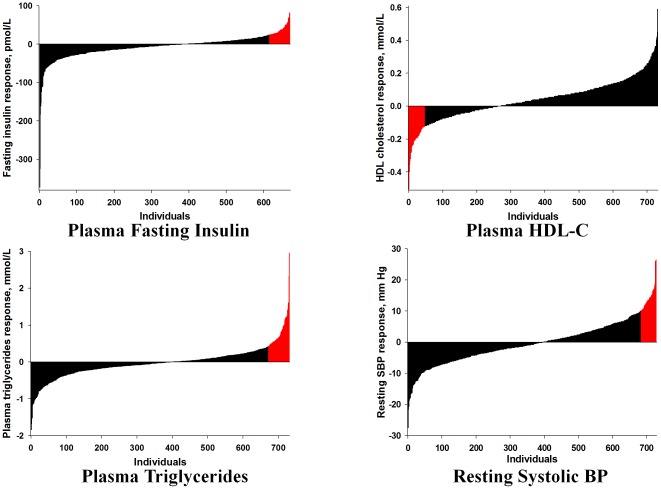
Distribution of the response to the HERITAGE exercise program with adverse responders highlighted in red. To convert pmol/L of insulin to mU/L, divide by 6.945. To convert mmol/L of HDL-C to mg/dL, divide by 0.02586. To convert mmol/L of triglycerides to mg/dl, divide by 0.01129.

Using the definitions outlined in Table 1, the prevalence of ARs for the four risk factors was first explored in the 473 Whites and 250 Blacks of HERITAGE who were all exposed to the same standardized exercise programs and were all qualified as completers. The results are depicted in Figure 1 and are summarized in Table 4. Although HERITAGE subjects were apparently healthy and not taking medication for blood pressure, glucose, or lipid anomalies and were exposed to the same exercise program, 6% to 9% of Blacks and Whites experienced ARs for each of the four risk factors, with no substantive differences between the two ethnic groups.

**Table 4:** Prevalence of Adverse Responders in HERITAGE.

		HERITAGE Whites (≤473)		HERITAGE Blacks (≤250)	
Risk factor	2×TE	N	%	N	%
Δ Fasting insulin	N≥24 pmol/L	38	9	17	9
Δ HDL-C	N≤0.12 mmol/L	28	6	19	8
Δ Triglycerides	N≥0.42 mmol/L	37	8	19	8
Δ Systolic BP	N≥10 mm Hg	28	6	16	7

To convert pmol/L of insulin to mU/L, divide by 6.945. To convert mmol/L of HDL-C to mg/dL, divide by 0.02586. To convert mmol/L of triglycerides to mg/dl, divide by 0.01129.

**Figure 2:**
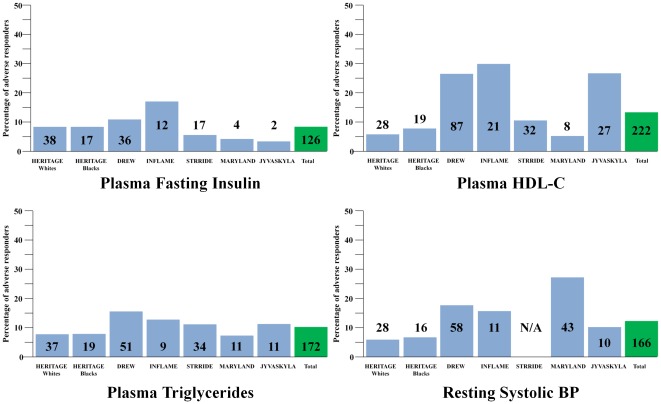
Percentages of adverse responders for each risk factor trait by study, with number of adverse responder subjects in each bar.

To gain a better understanding of the true prevalence of ARs for each risk factor, we compared the data obtained in HERITAGE with those of five other exercise training studies. The results are summarized in Table 5. It is quite obvious that the findings in HERITAGE are not unique to the HERITAGE subjects and exercise protocol. Based on a maximum of 1687 subjects, the prevalence of an AR reached 8.3% for the changes in FI, 13.3% for the changes in fasting HDL-C, 10.3% for TG, and 12.2% for resting SBP. The percentages of adverse responders for each trait for each study are depicted in Figure 2. It is remarkable that such cases were found in each study, even though the age and health status of the subjects were widely divergent and the exercise programs were quite heterogeneous.

**Table 5:** Prevalence of Adverse Responders to Regular Exercise in Six Studies.

	HERITAGE	DREW	INFLAME	STRRIDE	MARYLAND	JYVASKYLA	TOTAL	%*
N subjects	≤723	≤326	≤70	≤303	≤160	≤105	≤1687	
Δ Fasting insulin	55	36	12	17	4	2	126	8.3
Δ HDL-C	47	87	21	32	8	27	222	13.3
Δ Triglycerides	56	51	9	34	11	11	172	10.3
Δ Systolic BP	44	58	11	NA	43	10	166	12.2

* % represents the proportion of adverse responders in relation to the total number of subjects exercise trained for each of the four traits.

One important question to consider is whether those who respond adversely for a given risk factor are also those who experience the least improvement in cardiorespiratory fitness with regular exercise. This question was addressed by comparing the gains in VO_2_max between the subgroups of adverse responders and non-adverse responders for a given risk factor. The results of these analyses are shown in Table 6 for the gains in ml O_2_ per minute and the percentage increases in VO_2_max. A total of 56 differences were tested with age, sex, and baseline VO_2_max as covariates for the gain in ml O_2_ per minute and age and sex for the percentage increase. Only two such differences reached the 0.05 level of significance, and they were far from reaching a multiple test Bonferroni adjusted *P* value of 0.0009. These data indicate that AR traits are independent of the improvement in cardiorespiratory fitness.

**Table 6:** Comparison of the VO2max response to regular exercise between adverse responders and non-adverse responders for each response trait in each study.

	HERITAGE Whites		HERITAGE Blacks		DREW		INFLAME	
	Adverse responders	Non-adverse responders	Adverse responders	Non-adverse responders	Adverse responders	Non-adverse responders	Adverse responders	Non-adverse responders
**Δ Fasting insulin**								
N subjects	38	411	17	184	36	290	12	58
Δ VO_2_max (ml/min)	382 (34)	399 (10)	472 (43)	385 (14)	76 (22)	69 (7)	99 (61)	226 (28)
Δ VO_2_max (%)	16.1 (1.4)	17.0 (0.4)	20.6 (2.5)	18.3 (0.8)	6.0 (1.7)	5.8 (0.6)	8.0 (3.4)	14.5 (1.6)
**Δ HDL-C**								
N subjects	28	443	19	220	87	239	21	49
Δ VO_2_max (ml/min)	384 (40)	400 (10)	348 (39)	388 (12)	68 (14)	71(8)	219 (48)	196 (32)
Δ VO_2_max (%)	16.2 (1.7)	17.0 (0.4)	15.5 (2.3)	18.4 (0.7)	5.4 (1.1)	6.0 (0.7)	14.2 (2.7)	12.9 (1.8)
**Δ Triglycerides**								
N subjects	37	434	19	220	51	275	9	61
Δ VO_2_max (ml/min)	424 (34)	397 (10)	332 (39)	392 (13)	72 (18)	70 (8)	136 (72)	213 (28)
Δ VO_2_max (%)	17.7 (1.4)	16.9 (0.4)	16.9 (2.3)	18.3 (0.7)	6.1 (1.5)	5.8 (0.6)	8.6 (4.0)	14.0 (1.6)
**Δ Systolic BP**								
N subjects	28	442	16	220	58	268	11	59
Δ VO_2_max (ml/min)	348 (40)	401 (10)	396 (42)	386 (12)	60 (17)	72 (8)	140 (65)	215 (28)
Δ VO_2_max (%)	14.8 (1.7)	17.0 (0.4)	16.7 (2.5)	18.2 (0.7)	4.9 (1.4)	6.1 (0.6)	7.5 (3.6)	14.4 (1.6)

	STRRIDE		MARYLAND		JYVASKYLA	
	Adverse responders	Non-adverse responders	Adverse responders	Non-adverse responders	Adverse responders	Non-adverse responders
**Δ Fasting insulin**						
N subjects	17	286	4	92	2	57
Δ VO_2_max (ml/min)	278 (102)	278 (16)	112 (102)	306 (21)	340 (158)	277 (30)
Δ VO_2_max (%)	10.5 (3.6)	11.7 (0.7)	7.7 (5.0)	15.1 (1.0)	12.7 (8.8)	14.2 (1.6)
**Δ HDL-C**						
N subjects	32	271	8	142	26	71
Δ VO_2_max (ml/min)	231 (41)	281 (17)	206 (72)	274 (17)	183 (42)†	287 (26)
Δ VO_2_max (%)	11.4 (2.6)	11.6 (0.7)	10.0 (3.4)	13.4 (0.8)	8.1 (2.2)†	14.7 (1.3)
**Δ Triglycerides**						
N subjects	34	269	11	141	11	86
Δ VO_2_max (ml/min)	201 (46)	281 (17)	276 (62)	272 (17)	285 (67)	256 (24)
Δ VO_2_max (%)	8.3 (1.9)	12.1 (0.7)	13.4 (3.0)	13.3 (0.8)	12.3 (3.5)	13.0 (1.3)
**Δ Systolic BP**						
N subjects	N/A	N/A	43	115	10	87
Δ VO_2_max (ml/min)	N/A	N/A	230 (31)	271 (19)	244 (70)	261 (24)
Δ VO_2_max (%)	N/A	N/A	12.3 (1.5)	13.1 (0.9)	12.9 (3.7)	13.0 (1.2)

Data expressed as means and standard deviations.

Δ VO2max expressed as the change with exercise training in ml O2 per minute, reported as LS means with age, sex, and baseline VO2max as covariates. Δ VO2max % reported as LS means with age and sex as covariates.

† p≤0.05 indicates significant difference in VO2max training response between adverse responders and non-adverse responders.

One could hypothesize that the proportion of ARs should decrease as the amount of exercise increases. We tested this hypothesis with the data of DREW, and the results are summarized in Table 7. No substantive differences were observed in the prevalence of ARs among the three levels of exercise energy expenditure, which ranged from 4 to 12 kcal/kg of body weight per week.

**Table 7:** Adverse and Excellent Responders to Regular Exercise in DREW*.

		DREW4 kcal/kg/wk		DREW8 kcal/kg/wk		DREW12 kcal/kg/wk	
N subjects		143		89		94	
ADVERSE RESPONDERS		N	%	N	%	N	%
Δ Fasting insulin	N≥24 pmol/L	16	11	9	10	11	12
Δ HDL-C	N≤0.12 mmol/L	35	25	21	24	31	33
Δ Triglycerides	N≥0.42 mmol/L	19	13	14	16	18	19
Δ SBP	N≥10 mm Hg	32	22	14	16	12	

* A postmenopausal woman who follows the *2008 Physical Activity Guidelines for Americans* expends about 8 kcal/kg/week in her exercise program. The 4 kcal/kg/week is about 50% the current recommendation whereas the 12 kcal/kg/week is about 50% above the recommended dose.

Another important question is that of the proportion of subjects who experienced ARs for more than one risk factor. We tabulated the number of participants in the six studies who registered ARs for two or more risk factors, and the results are shown in Table 8. Approximately 7% of sedentary adults experienced ARs for at least two common cardiometabolic and diabetes risk factors following exposure to regular exercise. Only a small minority of participants (<1%) exhibited ARs for three or more traits.

**Table 8:** Percentage of Subjects in Each Study with 1, 2, or 3 and More Adverse Responses.

	1 Adverse Response		2 Adverse Responses		3 or 4 Adverse Responses	
	N	%	N	%	N	%
HERITAGE						
Blacks	51	20%	11	4%	0	0%
Whites	94	20%	17	4%	3	1%
DREW	131	40%	37	11%	9	3%
INFLAME	32	46%	9	13%	1	1%
STRRIDE	71	24%	9	3%	0	0%
MARYLAND	54	34%	5	3%	0	0%
JYVASKYLA	35	33%	7	7%	0	0%
TOTALS (mean %)	468	31%	95	6%	13	0.8%

The four traits considered were the exercise training-induced changes in fasting insulin, HDL-cholesterol, triglycerides, and resting systolic blood pressure.

## Discussion

The prevalence of ARs for select risk factors varied from 8.3% for the exercise training-induced changes in FI to 13.3% for the changes in HDL-C, with about 7% of participants experiencing adverse changes in two or more risk factors. This subgroup should receive urgent attention. The prevalence of ARs appears to be similar at low and high doses of exercise. However, we do not know whether some adverse responders would revert to a more positive response pattern if exposed to different exercise doses or exercise modalities.

It is important to differentiate between ARs for risk factors for common chronic diseases, as referred to in the present study, from other more acute ARs such as cardiac events related to exertion during an exercise bout [20], [21], [22], sudden cardiac death during or immediately after exercise typically associated with a cardiomyopathy or a congenital abnormality [23], or even exercise intolerance due to abnormal skeletal muscle energy metabolism [24]. These events are fortunately rare among physically active people. In contrast, ARs as defined herein for common cardiometabolic and diabetes risk factors are much more prevalent and become evident with exposure to regular exercise. It is not known whether such ARs can be detected after a single or a few bouts of exercise.

Even though the presence of ARs was first detected among completers in Blacks and Whites of the HERITAGE Family Study, in which subjects were confirmed to be sedentary at baseline, with a rather healthy profile, the phenomenon was confirmed in five other exercise intervention studies. The consistency in the prevalence of ARs across heterogeneous studies in terms of health status of subjects at baseline and of exercise training regimen is notable.

One question that may arise is whether ARs are the result of unwarranted exercise-drug interaction effects. The question cannot be answered with direct experimental data at the moment, but based on our analysis of the results of the six studies, it is highly unlikely that it is the case. For instance, HERITAGE and JYVASKYLA subjects were healthy adults taking no medication for high blood pressure, hypercholesterolemia, or hyperglycemia. However, many subjects in DREW, INFLAME, MARYLAND, and STRRIDE were taking medications for high blood pressure, hyperglycemia, or dyslipoproteinemia. Yet substantial numbers of subjects with or without medication in these cohorts experienced one or more ARs.

The challenge is now to investigate whether baseline predictors of ARs can be identified to screen individuals at risk so that they can be offered alternative approaches to modifying cardiometabolic risk factors. Research based on HERITAGE has amply demonstrated that the response pattern to exercise training aggregates in families [25], [26], [27], 28. In fact, the heritability of the changes induced by the exercise program reached about 30% for plasma HDL-C and TG [26] and about 20% to 25% for indicators of insulin metabolism and resting SBP [29], [30]. There are strong indications from a baseline skeletal muscle gene expression profile and from a genome-wide association study performed on the Whites of HERITAGE that the genetic component of a response trait can be defined in terms of RNA abundance observed in the sedentary state or by specific genomic variants [31], [32], [33]. This suggests that it may be possible with further research to identify molecular predictors of the inability to benefit from regular exercise and of adverse changes in specific cardiometabolic and diabetes risk factors.

In summary, we did not find any evidence for differences in the prevalence of ARs between Blacks and Whites or between men and women. Moreover, the AR traits are not explained by prior health status of subjects, age, amount of exercise imposed by the program, or lack of improvement in cardiorespiratory fitness. No evidence could be found for the hypothesis that ARs were the result of drug-exercise interactions. Thus, some individuals experience ARs when exposed to regular exercise, but the causes of the phenomenon are unknown at this time. The observations reported herein need to be extended to other cardiometabolic and diabetes risk factors such as LDL-cholesterol, small, dense LDL particles, markers of low-grade inflammation, adiposity traits, and ectopic fat depots. We conclude that it is critical to search for potential physiological and molecular predictors so that individuals at risk for adverse response patterns can be identified and offered proper guidance in an exercise medicine preventive or therapeutic context.

## Supporting Information

Information S1 **Detailed description of the six studies and the background material used to determine the technical error for fasting insulin.**
(DOCX)
