# Supplementary material for: Adverse Metabolic Response to Regular Exercise: Is It a Rare or Common Occurrence?
Source: PLoS One. 2012 May 30;7(5):e37887. doi: 10.1371/journal.pone.0037887 (PMC3364277; doi:10.1371/journal.pone.0037887)
Supplement: Information S1 — Detailed description of the six studies and the background material used to determine the technical error for fasting insulin. (DOCX) [file pone.0037887.s001.docx]

**SUPPORTING INFORMATION S1**

In support of this manuscript, this section contains a detailed description of the six studies and the background material used to determine the technical error for fasting insulin.

**Adverse Response to Regular Exercise: Is It a Rare or Common Occurrence?**

Claude Bouchard, Ph.D., Steven N. Blair, P.E.D., Timothy S. Church, M.D., M.P.H., Ph.D., Conrad P. Earnest, Ph.D., James M. Hagberg, Ph.D., Keijo Häkkinen, Ph.D., Nathan T. Jenkins, Ph.D., Laura Karavirta, Ph.D., William E. Kraus, M.D., Arthur S. Leon, M.S., M.D., D.C. Rao, Ph.D., Mark A. Sarzynski, Ph.D., James S. Skinner, Ph.D., Cris A. Slentz, Ph.D., and Tuomo Rankinen, Ph.D.

**Table of Contents**

Description of the Six Studies Pages 2-21

Estimate of the Technical Error for Fasting Plasma Insulin Page 22-24

References Pages 25-26

**DESCRIPTION OF THE SIX STUDIES**

**HERITAGE Family Study**

Full details of the Heritage Family Study design and methods can be obtained elsewhere [[1](#_ENREF_1)].

**Participants**

Recruitment of families was based on extensive publicity and advertisement. Family members were required to be sedentary at baseline (physical activity < 30 minutes on ≤ 1 day per week over the previous 6 months), between the ages of 17 and 65 years (17 to 41 years for offspring and 65 years or less for parents), free from preexisting disease, and normotensive or mildly hypertensive (< 160/100 mm Hg) without medications for hypertension, diabetes, or dyslipidemia. Overall, 834 participants from 218 families were recruited to participate in an endurance exercise training study and have measurements of baseline VO_2_max. Among them, 473 adults from 99 families of Caucasian descent completed the training and testing requirements. Likewise, 250 Blacks from 105 families or sibships were defined as completers. A completer was defined as having met at least 96% of all the exercise program requirements. Protocols were approved by each of the five participating centers’ Institutional Review Boards (IRBs), with written informed consent obtained from each participant. Subsequently, approval was obtained from the Pennington Biomedical Research Center’s IRB for continued analysis.

**Maximal Cardiorespiratory Capacity**

Two maximal exercise tests to measure VO_2_max were performed on two separate days at baseline and follow-up using a SensorMedics 800S (Yorba Linda, CA) cycle ergometer and SensorMedics 2900 metabolic measurement cart. The tests were conducted at about the same time of day, with at least 48 hours between the two tests. In the first test, participants exercised at a power output of 50 W for 3 minutes, followed by increases of 25 W every 2 minutes until volitional exhaustion. For older, smaller, or less fit individuals, the test was started at 40 W, with increases of 10–20 W every 2 minutes thereafter. In the second test, participants exercised for ~10 minutes at an absolute (50 W) and at a relative power output equivalent to 60% VO_2_max. They then exercised for 3 minutes at a relative power output that was 80% of their VO_2_max, after which resistance was increased to the highest power output attained in the first maximal test. If the participants were able to pedal after 2 minutes, power output was increased every 2 minutes thereafter until they reached volitional fatigue. The average VO_2_max from these two tests was taken as the VO_2_max for that subject and used in analyses if both values were within 5% of each other. If they differed by > 5%, the higher VO_2_max value was used.

**Resting Blood Pressure**

Blood pressure was taken using an automated blood pressure unit (Colin STBP-780; San Antonio, TX) following a 5-minute rest period. Four readings were taken at 2-minute intervals reported as the mean of the three or more reliable measurements.

**Venipuncture**

Blood samples were obtained from an antecubital vein into Vacutainer tubes containing EDTA in the morning after a 12-hour fast with participants in a semi-recumbent position. The blood samples were collected twice at baseline (on separate days) and again at 24 and 72 hours after the last training session. For eumenorrheic women, all samples were obtained in the early follicular phase of the menstrual cycle when blood plasma cholesterol alterations are minimal.

**Insulin, Triglyceride, and HDL-Cholesterol Assays**

Total cholesterol and triglyceride levels were determined in plasma and lipoproteins by enzymatic methods using a Technicon RA-500 Analyzer (Bayer Corporation Inc., Tarrytown, NY). The HDL-cholesterol (HDL-C) fraction was obtained after precipitation of LDL in the infranatant by the heparin manganese chloride method.

Fasting plasma insulin was obtained at baseline and 1 day and 3 days after the last exercise bout. Plasma insulin was measured by radioimmunoassay after polyethylene glycol separation [[2](#_ENREF_2)]. Polyclonal antibodies cross-react more than 90% with proinsulin [[3](#_ENREF_3)]. Therefore, in this study, insulin refers to immunoreactive insulin. In the present cohort with normal fasting glucose levels and no history of diabetes, it is estimated that about 10% of the immunoreactive insulin is in the form of proinsulin and its conversion intermediates [[4](#_ENREF_4)]. The intra- and inter-assay coefficients of variation were 7.7% and 10.3%, respectively.

**Training Program**

Participants exercised three times per week for 20 weeks on cycle ergometers. The intensity of the training was customized for each individual based on heart rate, and VO_2_ measurements were taken at the baseline. Briefly, participants trained at a heart rate associated with 55% of baseline VO_2_max for 30 minutes per session for the first 2 weeks. Thereafter, the duration or intensity was gradually increased every 2 weeks, until reaching 50 minutes at the heart rate associated with 75% of baseline VO_2_max. This level was maintained for the final 6 weeks of training. All training was performed on Universal Aerobicycles (Cedar Rapids, IA). Power output was controlled directly relative to heart rate by using the Universal Gym Mednet (Cedar Rapids, IA) computerized system. The protocol was standardized across all clinical centers and supervised to ensure that the equipment was working properly and that the participants were compliant with the protocol. Thus, compliance was 100% across all centers.

**Dose Response to Exercise in Women (DREW) Study**

Full details of the DREW study design and methods can be obtained elsewhere [[5](#_ENREF_5)].

**Participants**

Participants were recruited using a wide variety of techniques including newspaper, radio, television, mailers, community events, and e-mail distributions. A total of 4545 telephone screens were conducted. After giving informed consent, 464 postmenopausal women within the age range of 45 to 75 years who were sedentary (physical activity < 20 minutes on < 3 days per week, and < 8000 steps per day assessed over the course of 1 week) and overweight or obese (body mass index [BMI] 25.0 to 43.0 kg/m^2^) and had a systolic blood pressure of 120.0 to 159.9 mm Hg were randomized to one of three exercise groups or a non-exercise control. Exclusion criteria included history of stroke, heart attack, or any serious medical condition that prevented participants from adhering to the protocol or exercising safely. The study was originally reviewed annually by the IRB of the Cooper Institute and subsequently approved by the Pennington Biomedical Research Center’s IRB for continued analysis. Written informed consent was obtained from each participant.

**Maximal Cardiorespiratory Capacity**

Fitness testing was conducted using a Lode Excalibur Sport cycle ergometer (Groningen, Netherlands) using an electronically governed, pedal-rate-independent workload program. Participants initiated their testing protocol at 30 W for 2 minutes, moved to a steady-state stage at 50 W for 4 minutes, and then proceeded through the rest of their test by increasing workload 20 W every 2 minutes until exhaustion. Respiratory gases were measured using a ParvoMedics True Max 2400 metabolic measurement cart (Sandy, UT). Two fitness tests were performed on separate days at baseline and follow-up. The average VO_2_max from these two tests was used as VO_2_max.

**Resting Blood Pressure**

A minimum of four blood pressure measurements were made after a 30-minute recumbent rest period using an automated blood pressure unit (Colin STBP-780) with participants in the recumbent position. Each measure was separated by 2 minutes.

**Venipuncture**

The venipuncture was done in a fasting state (10-12 hours) at baseline and follow-up via an antecubital vein.

**Insulin, Triglyceride, and HDL-Cholesterol Assays**

Blood lipid profiles and fasting blood glucose concentration were determined via a Dimension RXL analyzer (Oxford, CT). Plasma insulin was measured by electrochemiluminescence.

**Training Program**

The exercise energy expenditure for women in the DREW study was designed to meet the consensus public health recommendation from the NIH and other organizations [[6](#_ENREF_6),[7](#_ENREF_7)]. Data from sedentary women recruited for previous exercise trials were used [[8](#_ENREF_8),[9](#_ENREF_9),[10](#_ENREF_10),[11](#_ENREF_11)]. Specifically, it was estimated that the typical sedentary postmenopausal woman who started an activity program and followed the consensus public health recommendation would expend 8 kcal/kg/week in the exercise program. Details of these calculations are presented in the DREW design and methods report [[5](#_ENREF_5)]. A major objective of DREW was to evaluate exercise levels 50% below and 50% above the public health recommendations to test whether the lower dose would provide any benefit and whether the higher dose would provide proportionally more benefit than the standard 8 kcal/kg/week exercise level. Thus, women were assigned to either a non-exercise control group or to groups that expended 4, 8, or 12 kcal/kg/week. Exercising women participated in three or four training sessions each week for 6 months, with training intensity at the heart rate associated with 50% of each woman's peak VO_2_.

All exercise sessions were performed under observation and supervision in an exercise laboratory with complete and strict monitoring of the amount of exercise completed in each session. Participants were weighed each week, and their weight was multiplied by their exercise dosage to determine the number of calories to be expended for the week. Women in the exercise groups alternated training sessions on semi-recumbent cycle ergometers and treadmills. Adherence to exercise training over the entire 6-month period was calculated for each individual by dividing the kilocalories expended during the exercise training by the kilocalories prescribed for the training period and multiplying by 100%.

Exercise adherence across randomization groups, defined as the percentage of expended calories with respect to prescribed calories for the entire 6-month intervention period, ranged from a low of 89.0% in the 8 kcal/kg/week group to a high of 94.6% in the 4 kcal/kg/week group when all participants were examined. The adherence increased to over 97% for all groups when only study completers were examined. Adherence rates did not differ significantly across treatment groups, age groups, or various racial/ethnic groups.

**Inflammation and Exercise (INFLAME) Study**

Full details of the INFLAME study design and methods can be obtained elsewhere [[12](#_ENREF_12)].

**Participants**

After giving written informed consent, 162 men and women between the ages of 30 and 75 who were sedentary (physical activity < 20 minutes on < 3 days per week) and who had an elevated plasma CRP concentration (≥ 2.0 mg/L but < 10.0 mg/L) at the initial screening were randomized an endurance group or a control group. Exclusion criteria included smoking and a history of stroke, diabetes, heart attack, BMI ≤18.5 or ≥ 40.0 kg/m^2^, or any serious medical condition that prevented participants from adhering to the protocol or exercising safely. Persons on beta-blockers or systemic steroids and women on hormone therapy were also excluded. The present report relies on data from 70 completers of the exercise group. The study was approved by the IRB of the Cooper Institute and reviewed quarterly by the IRB and a Data Safety and Monitoring Board.

**Maximal Cardiorespiratory Capacity**

Fitness testing was conducted using a Lode Excalibur Sport cycle ergometer and an electronically governed, pedal-rate-independent workload program. Participants initiated their testing protocol at 30 W for 2 minutes, moved to a steady-state stage at 50 W for 4 minutes, and then proceeded through the rest of their test by increasing workload 20 W every 2 minutes until exhaustion. Respiratory gases were measured using a ParvoMedics True Max 2400 metabolic measurement cart.

**Resting Blood Pressure**

A minimum of four blood pressure measurements were made after a 30-minute recumbent rest period using an automated blood pressure unit (Colin STBP-780) with participants in the recumbent position. Each measurement was separated by 2 minutes.

**Venipuncture**

Blood was drawn at three separate visits throughout the trial: 3 ml at the orientation session, 20 ml at baseline approximately 2 weeks later, and 20 ml at the 4-month follow-up assessment. Prior to all blood draws, participants fasted for 10–12 hours, refrained from consuming alcohol or exercising for 24 hours, and refrained from acutely using aspirin or anti-inflammatory medications for 48 hours.

**Insulin, Triglyceride, and HDL-Cholesterol Assays**

Blood lipid profiles and fasting blood glucose concentration were determined via a Dimension RXL analyzer. Plasma insulin was measured by electrochemiluminescence.

**Training Program**

The exercise dose was 16 kcal/kg/week divided into three to five sessions per week on a cycle ergometer or treadmill in a supervised exercise laboratory. This dose falls within the consensus public health recommendation for moderate- to vigorous-intensity physical activity of 30 minutes or more on most days of the week or approximately 150 to 210 minutes of moderate-intensity activity. For the purposes of this study, moderate- to vigorous-intensity exercise was defined as 60 to 80% of VO_2_max. All exercise sessions were performed under observation and supervision in an exercise laboratory, with strict monitoring of the amount of exercise completed in each session. Participants were weighed each week, and their weight was multiplied by 16 to determine the number of calories to be expended for the week. Exercise intensity was quantified using data from a Polar XL heart rate monitor (Lake Success, NY) worn by participants. The appropriate heart rate range for the prescribed intensity (60–80% VO_2_max) was calculated from the baseline maximal exercise test. If a participant’s heart rate fell or rose out of range during the intervention, the speed and/or grade of the treadmill or watts on the cycle ergometer were adjusted to maintain the prescribed intensity. Adherence to exercise training over the entire 4-month period was calculated for each individual by dividing the kilocalories expended during the exercise training by the kilocalories prescribed for the training period and multiplying by 100%.

For exercisers who completed the study, the mean and median exercise compliance was 91% and 99.9%, respectively. The mean exercise-training intensity was 75.3% (6.5 SD) of maximal heart rate, and the mean number of minutes per week spent exercising was 204 (45 SD) (excluding the ramp-up period).

**Studies of a Targeted Risk Reduction Intervention through Defined Exercise (STRRIDE) and STRRIDE Aerobic Training Versus Resistance Training (AT/RT)**

Full details of the STRRIDE and STRRIDE AT/RT study design and methods can be obtained elsewhere [[13](#_ENREF_13),[14](#_ENREF_14),[15](#_ENREF_15)].

**Participants**

Participants responded to local advertisements and were screened by phone. Inclusion criteria for STRRIDE were: age 40–65 years old, sedentary (physical activity < two times per week), BMI 25–35 kg/m^2^, with mild to moderate dyslipidemia (LDL-cholesterol 130–190 mg/dL and/or HDL-cholesterol < 40 mg/dL for men or <45 mg/dL for women). Exclusion criteria included individuals who smoked; had a history of diabetes, hypertension, or coronary artery disease; were currently dieting or intending to diet; had musculoskeletal limitations; or were not willing to be randomized to any group. Inclusion and exclusion criteria for STRRIDE AT/RT were identical except that the age range was increased to 18–70 years. After informed written consent, participants were randomized into one of the exercise training groups. Participants recruited for STRRIDE and STRRIDE AT/RT who were assigned to an aerobic exercise-only or a combination aerobic and resistance group (but not a resistance-only group) were included in the current analysis; 303 completers (146 women; 85% Whites) whose mean age was 51.0 ± 7.7 (SD) years were used. The study protocols were approved by the IRBs at Duke University Medical Center and East Carolina University.

**Maximal Cardiorespiratory Capacity**

A maximal cardiopulmonary exercise test with a 12-lead ECG and expired gas analysis was performed on a treadmill using a ParvoMedics TrueMax 2400 metabolic measurement cart twice at baseline and after completion of the exercise program. The protocol consisted of 2-minute stages, increasing the workload by approximately 1 MET per stage. The last 40 seconds were averaged to determine peak VO_2_.

**Resting Blood Pressure**

For STRRIDE, resting blood pressure was obtained in the standing position just prior to the maximal treadmill test. For STRRIDE AT/RT, resting blood pressure was taken at rest during the last hour of the 3-hour intravenous glucose tolerance test, approximately 20 minutes apart, and averaged.

**Venipuncture**

All blood samples were taken pre- and post-intervention, approximately 30 minutes before the start of the intravenous glucose tolerance test, in the fasted state. Post-intervention samples were taken 16 to 24 hours after the last exercise training session. The samples were taken from an intravenous catheter generally placed in the antecubital area. Blood samples were obtained using EDTA tubes, kept on ice, and spun within 30 minutes.

**Insulin, Triglyceride, and HDL-Cholesterol Assays**

Pre and post plasma samples from the same subject were analyzed together to control for inter-technician and inter-assay variability. Fasting insulin was determined with immunoassay (Access Immunoassay System, Beckman Coulter, Fullerton, CA). Fasting HDL-C and triglyceride concentrations were estimated via NMR spectroscopy (LipoScience, Raleigh, NC).

**Training Program**

The exercise training duration was 8 months (approximately 35 weeks), and the exercise prescription for the groups from STRRIDE and STRRIDE AT/RT included: (1) low amount/moderate intensity (equivalent to 14 kcal/kg/week) at 50% of peak VO_2_; (2) low amount/vigorous intensity (14 kcal/kg/week) at 75% of peak VO_2_; (3) high amount/vigorous intensity (23 kcal/kg/week) at 75% of peak VO_2_; and (4) AT/RT equivalent to the low amount/vigorous intensity (group 2 above) plus resistance training (3 days per week, 8 exercises, 3 sets per exercise, 8–12 repetitions per set).

All aerobic exercise sessions were verified with downloadable heart rate monitors for both intensity and duration. Resistance training was verified either by supervising personally or electronically by the FitLinxx Strength Training Partner™ system (Norwalk, CT). The average duration and frequency for each group was [mean (SD)]: (1) low amount/moderate intensity was 3.6 (0.5) sessions per week and 179 (37) minutes per week; (2) low amount/vigorous intensity was 3.0 (0.5) sessions per week and 125 (25) minutes per week; (3) high amount/vigorous intensity was 3.7 (0.8) sessions per week and 181.4 (39) minutes per week; and (4) AT/RT was 2.9 (0.6) sessions per week and 133 (25) minutes per week of aerobic exercise and 2.5 (0.4) sessions per week and an estimated 157.5 minutes per week of resistance training.

**University of Maryland Gene Exercise Research Study**

Full details of the University of Maryland Gene Exercise Research Study design and methods can be obtained elsewhere [[16](#_ENREF_16)].

**Participants**

One hundred and sixty men and women aged 50 to 75 years were recruited via media advertisements, direct mail, and personal contacts and screened via telephone. Participants were sedentary (physical activity < 20 minutes < two times per week and being retired or with a sedentary occupation), normotensive or with blood pressure controlled with medications not affecting plasma glucose or lipid levels, nondiabetic and nonsmoking, with no prior history of cardiovascular disease and BMI <37 kg/m^2^. Participants had to have one NCEP lipid abnormality or blood pressure in the prehypertensive or Stage 1 hypertension range. All women were postmenopausal and kept their hormone replacement therapy constant for the duration of the study (either on or not on HRT). Participants also had to have a negative screening maximal exercise test. The study was approved by the IRB at the University of Maryland, College Park, and all participants provided their written consent during their first laboratory visit.

**Maximal Cardiorespiratory Capacity**

VO_2_max was measured using a modified Bruce exercise test to exhaustion before and after exercise training. For the post-training test, the initial treadmill speed was set to elicit 70% of baseline VO_2_max, while the incline was increased every 2 minutes until exhaustion.

**Resting Blood Pressure**

Participants came to the laboratory fasted and sat in a quiet, thermoneutral room for 15 minutes prior to the blood pressure measurements. Blood pressure was then measured while seated with the arm at heart level. Measurements were taken until two values agreed within + 5 mmHg. Measures were taken on 2 days, and the averages from the 2 days were averaged to determine the final blood pressure.

**Venipuncture**

Venous blood samples were drawn in the morning, with the participants having undergone an overnight fast. Blood samples for plasma lipid levels were drawn on two separate days and averaged to derive the final value. Blood samples before training were drawn after completion of the 6-week dietary stabilization period and at least 3 days after any prior exercise testing. After training, samples were drawn 24 to 36 hours after the participant’s last exercise session.

**Insulin, Triglyceride, and HDL-Cholesterol Assays**

Plasma HDL-C levels were measured on a CDC-certified Hitachi 717 analyzer after precipitation with dextran sulfate. Triglycerides were also measured on the same analyzer. Plasma insulin levels were measured via radioimmunoassay (Linco Research, St. Charles, MO).

**Training Program**

Training began with 1 week of three 20-minute sessions at a heart rate-monitored intensity of 50% of baseline VO_2_max. Duration then increased by 5% VO_2_max per week until 70% VO_2_max was achieved. At that point, training session duration was increased by 5 minutes per week until 40 minutes of exercise was achieved per session. These training sessions utilized treadmills, elliptical, rowing, and cross-country ski ergometers. Training intensity was monitored with a wrist heart rate monitor. After 12 weeks of training, a lower intensity 45- to 60-minute walk at home was added on the weekend. Compliance was assessed by attendance and achieving the prescribed intensity and duration for each training session. Only participants achieving > 75% compliance were included in the final data set. Also, participants were included in the final data set only if they did not lose more weight with the exercise intervention than would have been expected based on their energy expenditure. Participants were also monitored to ensure that they maintained an AHA Step 1 diet during an initial 6-week dietary stabilization phase and then for the duration of the exercise training intervention.

**University of Jyväskylä Study**

Full details of the University of Jyväskylä study design and methods can be obtained elsewhere [[17](#_ENREF_17)].

**Participants**

Healthy, untrained 40- to 67-year-old men and women were recruited by advertising in newspapers and through e-mail lists. Participants had been previously involved with various recreational physical activities such as walking, jogging, cross-country skiing, aerobics, or biking, but none of the participants had any background in regular strength training or competitive sports. Participants underwent a general health examination, a recording of resting ECG and a clinical exercise test to voluntary exhaustion with ECG monitoring. Exclusion criteria were cardiovascular disease, musculoskeletal problems, diabetes, or any other disease that may have prevented performing the requested testing or training, medications known to influence cardiovascular or neuromuscular performance, and systematic endurance or strength training background during the year preceding the study. The participants were informed about the design of the study and possible risks and discomforts related to the measurements, after which all participants signed a written informed consent. The Ethics Committee of the University of Jyväskylä approved the study plan.

A total of 206 participants entered the study and were randomized into an endurance training group (E, N=51) and a combined endurance and strength training group (ES, N=59). The other participants, who are not included in the present study design, performed strength training or served as controls. Five participants dropped out in the ES group due to musculoskeletal injuries, cardiac problems, or personal reasons. Finally, data for the E and ES groups were pooled and analyzed for 25 men and 26 women in the E group and 30 men and 24 women in the ES group. They were completers who met 90% or more of the program requirements.

**Maximal Cardiorespiratory Capacity**

A graded maximal exercise test to volitional fatigue was performed on a mechanically braked bicycle ergometer (Ergomedic 839E, Monark Exercise AB, Sweden) with simultaneous ECG and blood pressure monitoring both before and after exercise training. The test was supervised by a physician, started at 50 W, and was then increased by 20 W every 2 minutes throughout the test. Participants maintained a pedalling frequency of 60 rpm throughout the test and were verbally encouraged to maintain the required pedalling frequency as long as possible. Oxygen uptake (VO_2_), carbon dioxide production (VCO_2_), ventilation, breathing frequency, and other standard respiratory parameters were measured continuously breath by breath (SensorMedics Vmax229). VO_2_max was determined as the highest minute average of VO_2_ during the test.

**Resting Blood Pressure**

Systolic blood pressure was recorded as the lowest of two measurements in a supine position after a rest of 5 minutes using an automatic sphygmomanometer (Omron, model HEM-705C, Omron Corporation, Hamburg, Germany).

**Venipuncture**

All blood samples were taken after a 12-hour fast between 7 A.M. and 9 A.M. Participants were instructed to avoid strenuous physical activity on the preceding day and to rest at least 8 hours during the previous night. All blood samples were drawn from the antecubital vein and handled according to standardized laboratory practice.

**Insulin, Triglyceride, and HDL-Cholesterol Assays**

Insulin concentrations were assayed using TR-IFMA (time-resolved immune-fluorometric assays, B080-101) and an AutoDELFIA fluorometer (Wallac, Turku, Finland). HDL-C and triglycerides were determined using Vitros DT60 dry chemistry system (Ortho-Clinical Diagnostics, Inc., USA) in women and the enzymatic assays (micro-flow spectrophotometer Shimadzu CL-720, Kyoto, Japan) and kits from Roche Diagnostics GmbH (Mannheim, Germany) in men.

**Training Program**

Endurance training was carried out twice a week for 21 weeks. Heart rate levels for endurance training were determined using respiratory parameters and blood lactate concentrations based on the results from two VO_2_max tests performed at baseline [[18](#_ENREF_18)]. All training sessions were supervised, and heart rate was monitored. During the first 7 weeks, participants trained on a bicycle ergometer for 30 minutes mainly below the level of the aerobic threshold. During weeks 8 to 14, one weekly session of 45 minutes included a 10-minute interval between the aerobic-anaerobic thresholds and a 5-minute interval above the anaerobic threshold, in addition to a 15-minute warm-up and a 15-minute cooldown. The other weekly training session involved 60 minutes of cycling below the aerobic threshold. During the last training weeks of 15 to 21, one of the weekly sessions lasted for 60 minutes, which included two 10-minute intervals between the aerobic-anaerobic thresholds, two 5-minute intervals above the anaerobic threshold, and a total of 30 minutes below the aerobic threshold performed before, after, and between the intervals. The other weekly session included 90 minutes of cycling at a steady pace below the aerobic threshold.

The ES group performed combined endurance and strength training, i.e., strength training twice a week in addition to the previously described endurance training program, performing a total of four training sessions per week. All strength training sessions were supervised, and training diaries were used to monitor training volume and intensity. Every training session included 2–4 sets of 7–10 exercises, 2 of which were for the leg extensors (leg press and knee extension), 1 for the knee flexors (leg curl), and 1 to 2 other exercises for the lower extremities (seated calf raise, hip abduction or adduction). For the upper body, each session included 3 to 4 exercises (bench press, biceps curl, triceps pull-down, lateral pull-down), and 1 to 2 exercises were for the trunk (abdominal crunch, back extension). The overall training intensity increased progressively throughout the 21-week training period from 40–60% of one repetition maximum (1RM) and 12–20 repetitions to 70–85 % of 1RM and 5–8 repetitions.

**ESTIMATE OF THE TECHNICAL ERROR FOR FASTING PLASMA INSULIN**

The technical error (TE) is defined as the within-subject standard deviation (SD) as derived from repeated measures (or assays) over a given period of time. An ancillary study designed to quantify TE for several biological traits was undertaken in HERITAGE. Sixty subjects were measured three times (except for fasting insulin) over a period of 3 weeks for each trait [[19](#_ENREF_19),[20](#_ENREF_20),[21](#_ENREF_21),[22](#_ENREF_22),[23](#_ENREF_23)]. The insulin measurements were performed only twice over the 3-week period using two different assays, a sensitive assay and a simple clinical diagnostic assay. However, we also had repeated measures of fasting insulin on 779 HERITAGE subjects at baseline (prior to the exercise program) and on 624 participants after the 20-week program.

Fasting plasma insulin was obtained at baseline and 1 day and 3 days after the last exercise bout in HERITAGE participants. Plasma insulin was measured by radioimmunoassay after polyethylene glycol separation [[2](#_ENREF_2)]. Polyclonal antibodies cross-react more than 90% with proinsulin [[3](#_ENREF_3)]. Therefore, in this study, insulin refers to immunoreactive insulin. It is estimated that about 10% of the immunoreactive insulin is in the form of proinsulin and its conversion intermediates [[4](#_ENREF_4)]. The intra- and inter-assay coefficients of variation were 7.7% and 10.3%, respectively.

We used the data from these three HERITAGE sources plus observations from the literature to develop an estimate of TE for fasting insulin. The HERITAGE results are summarized here.

- Results from the 60 subjects in which fasting insulin was assayed twice:
- Intraclass correlation (ICC)=0.32
- TE=18
- Coefficient of variation (CV)=34.7%
- Pretraining results based on two fasting insulin assays (N=779):
- ICC=0.94
- TE=13
- CV=18.8%
- Post-training results based on two fasting insulin assays (N=624):
- ICC=0.78
- TE=20
- CV=29.5%

The most frequently reported parameter of within-individual variation in studies that have considered the variation in fasting insulin level is the CV. In a recent study on within-subject variability, the CV for fasting insulin reached 19% in normoglycemic individuals, 14% in impaired glucose tolerant subjects, and 17% in diabetics with or without medication [[24](#_ENREF_24)]. In eight subjects who were measured five times over 17 months, the CV reached 35% [[25](#_ENREF_25)]. In 12 healthy adults of both sexes who were assessed on 12 consecutive days, the CV for fasting insulin reached 26% [[26](#_ENREF_26)]. All these CV values compare well with those reported above for the various HERITAGE data sets.

We assumed that about 10% of the intra-individual differences was due to the assay error variance. We have a range of CVs (from 14% to 35%) across all studies and of TEs (from 13 pmol/L to 20 pmol/L) across HERITAGE data sets. With a mean fasting level of about 66 pmol/L at baseline and a CV of 30% as we observed in HERITAGE, we hypothesized that a decrease of about 20 pmol/L would be sufficient to be indicative of an adverse response. We have therefore opted for the use of a conservative TE of 12 pmol/L and thus 24 pmol/L for 2 TEs for the present report.

**REFERENCES-SUPPORTING INFORMATION**

1. Bouchard C, Leon AS, Rao DC, Skinner JS, Wilmore JH et al (1995) The HERITAGE family study. Aims, design, and measurement protocol. Med Sci Sports Exerc 27: 721-729.

2. Desbuquo B, Aurbach GD (1971) Use of polyethylene glycol to separate free and antibody-bound peptide hormones in radioimmunoassays. J Clin Endocr Metab 33: 732-738.

3. Roder ME, Porte D, Schwartz RS, Kahn SE (1998) Disproportionately elevated proinsulin levels reflect the degree of impaired B cell secretory capacity in patients with noninsulin-dependent diabetes mellitus. J Clin Endocr Metab 83: 604-608.

4. Kahn SE, Leonetti DL, Prigeon RL, Boyko EJ, Bergstrom RW et al (1995) Relationship of proinsulin and insulin with noninsulin-dependent diabetes-mellitus and coronary heart-disease in Japanese-American men - Impact of obesity - Clinical research-center study. J Clin Endocr Metab 80: 1399-1406.

5. Morss GM, Jordan AN, Skinner JS, Dunn AL, Church TS et al (2004) Dose response to exercise in women aged 45-75 yr (DREW): Design and rationale. Med Sci Sports Exerc 36: 336-344.

6. NIH Consensus Development Panel on Physical Activity and Cardiovascular Health (1996) Physical activity and cardiovascular health. JAMA 276: 241-246.

7. U.S. Department of Health and Human Services (1996) Physical activity and health: A report of the Surgeon General. Atlanta, GA: U.S. Department of Health and Human Services, Centers for Disease Control and Prevention, National Center for Chronic Disease Prevention and Health Promotion.

8. Blair SN, Kohl HW, 3rd, Barlow CE, Paffenbarger RS, Jr., Gibbons LW et al (1995) Changes in physical fitness and all-cause mortality. A prospective study of healthy and unhealthy men. JAMA 273: 1093-1098.

9. Blair SN, Kohl HW, 3rd, Paffenbarger RS, Jr., Clark DG, Cooper KH et al (1989) Physical fitness and all-cause mortality. A prospective study of healthy men and women. JAMA 262: 2395-2401.

10. Dunn AL, Garcia ME, Marcus BH, Kampert JB, Kohl HW et al (1998) Six-month physical activity and fitness changes in Project Active, a randomized trial. Med Sci Sports Exerc 30: 1076-1083.

11. Dunn AL, Marcus BH, Kampert JB, Garcia ME, Kohl HW, 3rd, et al (1997) Reduction in cardiovascular disease risk factors: 6-month results from Project Active. Prev Med 26: 883-892.

12. Thompson AM, Mikus CR, Rodarte RQ, Distefano B, Priest EL et al (2008) Inflammation and exercise (INFLAME): Study rationale, design, and methods. Contemp Clin Trials 29: 418-427.

13. Bateman LA, Slentz CA, Willis LH, Shields AT, Piner LW et al (2011) Comparison of aerobic versus resistance exercise training effects on metabolic syndrome (from the Studies of a Targeted Risk Reduction Intervention Through Defined Exercise - STRRIDE-AT/RT). Am J Cardiol 108: 838-844.

14. Kraus WE, Torgan CE, Duscha BD, Norris J, Brown SA et al (2001) Studies of a targeted risk reduction intervention through defined exercise (STRRIDE). Med Sci Sports Exerc 33: 1774-1784.

15. Slentz CA, Bateman LA, Willis LH, Shields AT, Tanner CJ et al (2011) Effects of aerobic vs. resistance training on visceral and liver fat stores, liver enzymes, and insulin resistance by HOMA in overweight adults from STRRIDE AT/RT. Am J Physiol Endocrinol Metab 301: E1033-1039.

16. Wilund KR, Ferrell RE, Phares DA, Goldberg AP, Hagberg JM (2002) Changes in high-density lipoprotein-cholesterol subfractions with exercise training may be dependent on cholesteryl ester transfer protein (CETP) genotype. Metabolism 51: 774-778.

17. Karavirta L, Hakkinen K, Kauhanen A, Arija-Blazquez A, Sillanpaa E et al (2011) Individual responses to combined endurance and strength training in older adults. Med Sci Sports Exerc 43: 484-490.

18. Aunola S, Rusko H (1984) Reproducibility of aerobic and anaerobic thresholds in 20-50 year old men. Eur J Appl Physiol Occup Physiol 53: 260-266.

19. Gagnon J, Province MA, Bouchard C, Leon AS, Skinner JS et al (1996) The HERITAGE Family Study: Quality assurance and quality control. Ann Epidemiol 6: 520-529.

20. Wilmore JH, Stanforth PR, Domenick MA, Gagnon J, Daw EW et al (1997) Reproducibility of anthropometric and body composition measurements: The HERITAGE Family Study. Int J Obes Relat Metab Disord 21: 297-303.

21. Wilmore JH, Stanforth PR, Turley KR, Gagnon J, Daw EW et al (1998) Reproducibility of cardiovascular, respiratory, and metabolic responses to submaximal exercise: The HERITAGE Family Study. Med Sci Sports Exerc 30: 259-265.

22. Despres JP, Gagnon J, Bergeron J, Couillard C, Leon AS et al (1999) Plasma post-heparin lipase activities in the HERITAGE Family Study: the reproducibility, gender differences, and associations with lipoprotein levels. HEalth, RIsk factors, exercise Training and GEnetics. Clin Biochem 32: 157-165.

23. Skinner JS, Wilmore KM, Jaskolska A, Jaskolski A, Daw EW et al (1999) Reproducibility of maximal exercise test data in the HERITAGE family study. Med Sci Sports Exerc 31: 1623-1628.

24. Utzschneider KM, Prigeon RL, Tong J, Gerchman F, Carr DB et al (2007) Within-subject variability of measures of beta cell function derived from a 2 h OGTT: Implications for research studies. Diabetologia 50: 2516-2525.

25. Gravholt CH, Holck P, Nyholm B, Christiansen E, Erlandsen M et al (2000) No seasonal variation of insulin sensitivity and glucose effectiveness in men. Metabolism 49: 32-38.

26. Widjaja A, Morris RJ, Levy JC, Frayn KN, Manley SE et al (1999) Within- and between-subject variation in commonly measured anthropometric and biochemical variables. Clin Chem 45: 561-566.
